# Case report: Spinal anesthesia for cesarean section in a parturient with Potocki-Lupski syndrome

**DOI:** 10.1186/s12871-021-01433-3

**Published:** 2021-09-07

**Authors:** Junfeng Li, Jie Bao, Di Zhang, Shuzhen Zhou

**Affiliations:** Department of Anesthesiology, Beijing Hospital, National Center of Gerontology, Institute of Geriatric Medicine, Chinese Academy of Medical Science, No.1 DaHua Road, DongDan, Beijing, 100730 China

**Keywords:** Cesarean section, Potocki -Lupski syndrome, Spinal anesthesia, Case report

## Abstract

**Background:**

Potocki -Lupski syndrome is an uncommon disorder caused by a micro-duplication in chromosome 17p11.2. Variable clinical manifestations bring troubles to the general and neuraxial anesthesia, including mental retardation, facial dysmorphisms, structural cardiovascular anomalies, scoliosis, and malignant hyperthermia. Until now, the anesthesia management for cesarean section in these patients has not been reported yet.

**Case presentation:**

Here we present a 23-year-old Chinese parturient with Potocki -Lupski syndrome who underwent elective cesarean section under spinal anesthesia. She was transferred to our hospital in her 40th week of gestation. She had a history of IgA nephropathy for more than three years and was diagnosed with Potocki -Lupski syndrome (17p12p11.2 segment 3.1 Mb repeat) in the 29th week of pregnancy. Amniocentesis showed the fetus had no abnormal autosomes. Preoperative multidisciplinary consultation suggested that she should terminate the pregnancy as soon as possible. She was ASA II. Her BMI was 26.43 kg/m^2^. Her airway evaluation was normal. Her spine could bend well and her spinal interspace could be touched clearly. We did the single spinal anesthesia at L2-3 interspace and gave 0.5% bupivacaine 1.7 ml. The absolute anesthesia level reached T8. The Apgar score for the newborn infant was 10 for 1st minute, 5th minute, and 10th minute. The vital signs were steady without using any vasoactive drugs. The patient had a good prognosis, and was subsequently discharged from hospital.

**Conclusion:**

To date, the case may be the first reported spinal anesthesia for the parturient with Potocki -Lupski syndrome. Although its manifestations are variable, the spinal anesthesia is feasible under careful and comprehensive preoperative evaluation.

## Background

Potocki -Lupski syndrome (PTLS) is a rare disorder, with an incidence of approximately 1 in 25,000 live births. It is caused by a micro-duplication in chromosome 17p11.2, usually with a length of 3.7 Mb [1]. It is clinically recognizable and is associated with infantile hypotonia, failure to thrive, mental retardation and autistic features [2]. Those patients with PTLS are prone to difficult airway because of facial dysmorphisms and obstructive sleep apnea. Furthermore, structural cardiovascular anomalies, malignant hyperthermia and scoliosis may be the challenges for both general and neuraxial anesthesia. Up to now, only one report concerning general anesthetic management for a 21-month-old girl has been previously described [3]. We report here a parturient with PTLS who underwent a successful cesarean section under spinal anesthesia. Anesthetic management and some pertinent points are discussed.

## Case presentation

A 23-year-old Chinese primigravida with PTLS was transferred to the hospital in her 40th week of gestation, owing to facial edema and blurred vision for two days. In the 17th week of pregnancy, she was diagnosed with PTLS (17p12p11.2 segment 3.1 Mb repeat) by testing her peripheral blood chromosome, but amniocentesis showed the fetus had no abnormal autosomes in the 29th week of pregnancy. Her past medical history included IgA nephropathy and she denied a history of obstructive sleep apnea, past anesthesia and medications. She was 155 cm tall and 63.5 kg weighted. She presented speech delay, intellectual disability, and behavioral disturbances. Physical examination showed a blood pressure of 120/80 mmHg, a heart rate of 80/min, a respiratory rate of 18/min, and a temperature of 36.0℃. Her heart rate was regular in rate and rhythm. Lung fields were clear to auscultation. She had peripheral facial paralysis and anhidrosis in the right face. Her neurological reflexes and muscular strength were normal. She could bend her spine well and her lumbar intervertebral spaces were palpable. Her airway evaluations were normal and the modified Mallampati classification was II. Electrocardiography showed sinus rhythm at a rate of 100/min. Transthoracic echocardiography demonstrated no abnormalities in cardiac structure and function. Urinary ultrasound scan showed that the left kidney was 12.5*4.8 cm and the right kidney was 12.3*5.5 cm. Obstetrical ultrasound scan revealed that the fetus was normally developed and in a transverse lie. The laboratory results were normal with the exception of a serum creatinine of 94 μmol/L (normal value, 45–84 μmol/L) and a 24-h urine protein quantitation of 2.93 g (normal value, 0.028–0.141 g). There was a preoperative multidisciplinary consultation, including nephrologist, neurologist, otolaryngologist. The neurologist consulted that she has right peripheral facial paralysis, her right eyelid could not close totally, her limbs could move freely, her muscle strength was V, her neural reflexes were normal and bilateral pathological signs were negative. The otolaryngologist consulted that the pharynx was slightly congested, and the tongue was not skewed. The nephrologist consulted that she should terminate the pregnancy as soon as possible because of elevated creatinine. Since her cervix was not favorable, the decision for caesarean section was made by the obstetrician. We planned to administer spinal anesthesia with a backup plan of general anesthesia for elective cesarean section.

Routine non-invasive monitoring was established, including non-invasive blood pressure, heart rate, pulse oximetry, and electrocardiography. We prepared video laryngoscope and general anesthetics (propofol, sufentanil and rocuronium). We decided not to use depolarizing muscle relaxants and inhaled anesthetics for avoidance of malignant hyperthermia-triggering agents.The patient was placed in the lateral decubitus position after intravenous access was obtained. Three milliliters of 1% lidocaine was used for local infiltration anesthesia at L2-3 interspace. A 18G 3.5 cm syringe needle was used as the guiding. Then a 25-gauge pencil-point spinal needle was inserted via it. After cerebrospinal fluid was detected, 1.7 ml 0.5% hypobaric bupivacaine was given into the subarachnoid cavity, resulting in a bilateral T8 sensory level by blunt pinprick five minutes later. The Apgar score for the newborn infant was 10 at 1st, 5th, and 10th minute. A total of 1100 mL solution was infused to maintain adequate fluid status. Her intraoperative vital signs were stable without using any vasoactive drugs. Postoperative pain control was maintained by PCIA (Patient-Controlled-Intravenous-Analgesia) Apparatus with morphine at a rate of 1 mg/h for 48 h. No complications of anesthesia were observed. The patient was discharged uneventfully on postoperative day 3. Three months later, the patient and her family were followed up by phone and satisfied with the medical service. Written informed consent was obtained from the patient’s legal husband for publication of this case report.

## Discussion and conclusions

The clinical features of patients with PTLS are variable [4]. It is important to underline that some of them may bring troubles to the anesthesia. And early referral and multidisciplinary assessment are very important. Firstly, tracheal intubation or insertion of laryngeal mask airway may be difficult because of facial dysmorphisms and obstructive sleep apnea. These dysmorphisms are present in 43.1% of patients, such as down-slanting palpebral fissures, frontal bossing, low-set and posteriorly rotated ears, and broad mouth. Neira-Fresneda recommended otolaryngological evaluation to assess anatomy causes of obstructive sleep apnea [5]. Secondly, preoperative cardiovascular examination is necessary. Soler-Alfonso found 10 cardiovascular anomalies in 24 patients [6]. Aortic dilation has been reported in patients with PTLS, even without a history of heart defect or cardiothoracic surgery [6]. Echocardiographic screening and electrocardiographic exam were recommended at diagnosis with attention to the aortic root and great vessels. If abnormal, consult a cardiologist. If normal, reevaluate periodically after normal echocardiogram every 2–3 years in childhood and adolescence, every 4–5 years in adulthood for aortic root dilation [5]. In our case, the patient was diagnosed with PLTS during pregnancy and was transferred to our hospital on the weekend so that we could not do multidisciplinary assessment earlier in the antenatal management. Since the medical condition there was limited and the referral system has not been well established in China, she was not taken seriously at first. However, as she was admitted to our hospital, we arranged a multidisciplinary consultation including nephrologist, neurologist, otolaryngologist. And the routine preoperative airway evaluations and cardiovascular examinations were normal. Anesthesiologists can use ultrasound technology for more comprehensive assessments in the future.

We found only one case was reported by Fernández Urbón about the anesthetic management of a child with PTLS [3]. The child was a 21-month-old girl who presented with ogival palate, retrognathia, broad filtrum, axial hypotonia and hypertonia in lower extremities. She was intervened for inguinal hernia and underwent general anesthesia with propofol combined with ilioinguinal and iliohypogastric nerve block. She was discharged the next day. We agreed with Fernández Urbón’s advocation that anesthesiologists should avoid using drugs which could cause malignant hyperthermia, such as depolarizing muscle relaxants and inhaled anesthetics. In our case, we prepared propofol and rocuronium which had specific antagonists. Furthermore, neuraxial anesthesia may be difficult in the patients of PTLS. On the one hand, poor communication and cooperation may predispose a patient`s inability to maintain stillness during needle puncture and neuraxial anesthesia may not be administered successfully. On the other hand, scoliosis may increase the difficulty of puncture technique or risk of complication [7]. In our case, fortunately, spinal anesthesia was successfully performed at the first attempt without perioperative complications. If not, recent studies have shown that spinal ultrasound can be used for difficult puncture [8].

To date, the case may be the first reported spinal anesthesia for the parturient with PTLS. Careful and comprehensive preoperative evaluation is very important for patients with Potocki -Lupski syndrome during to the varieties of clinical manifestations. In the future, we not only need more cases to explore whether spinal anesthesia is safe and effective for cesarean section, but also we need to investigate whether general anesthesia is suitable for parturients with PTLS.

## Data Availability

All data and materials described in the manuscript will be freely available to any scientist wishing to use them for non-commercial purposes. SZZ should be contacted if someone wants to request the data.

## Abbreviations

PTLS: Potocki -Lupski syndrome
ASA: American society of Anesthesiologists
BMI: Body Mass Index
PCIA: Patient-Controlled-Intravenous-Analgesia
